# Independent Component Analysis of Gait-Related Movement Artifact Recorded using EEG Electrodes during Treadmill Walking

**DOI:** 10.3389/fnhum.2015.00639

**Published:** 2015-12-01

**Authors:** Kristine L. Snyder, Julia E. Kline, Helen J. Huang, Daniel P. Ferris

**Affiliations:** ^1^School of Kinesiology, University of MichiganAnn Arbor, MI, USA; ^2^Department of Mathematics and Statistics, University of Minnesota DuluthDuluth, MN, USA; ^3^Department of Biomedical Engineering, University of MichiganAnn Arbor, MI, USA

**Keywords:** walking, cortical sources, blind source separation, artifact removal, electroencephalography

## Abstract

There has been a recent surge in the use of electroencephalography (EEG) as a tool for mobile brain imaging due to its portability and fine time resolution. When EEG is combined with independent component analysis (ICA) and source localization techniques, it can model electrocortical activity as arising from temporally independent signals located in spatially distinct cortical areas. However, for mobile tasks, it is not clear how movement artifacts influence ICA and source localization. We devised a novel method to collect pure movement artifact data (devoid of any electrophysiological signals) with a 256-channel EEG system. We first blocked true electrocortical activity using a silicone swim cap. Over the silicone layer, we placed a simulated scalp with electrical properties similar to real human scalp. We collected EEG movement artifact signals from ten healthy, young subjects wearing this setup as they walked on a treadmill at speeds from 0.4–1.6 m/s. We performed ICA and dipole fitting on the EEG movement artifact data to quantify how accurately these methods would identify the artifact signals as non-neural. ICA and dipole fitting accurately localized 99% of the independent components in non-neural locations or lacked dipolar characteristics. The remaining 1% of sources had locations within the brain volume and low residual variances, but had topographical maps, power spectra, time courses, and event related spectral perturbations typical of non-neural sources. Caution should be exercised when interpreting ICA for data that includes semi-periodic artifacts including artifact arising from human walking. Alternative methods are needed for the identification and separation of movement artifact in mobile EEG signals, especially methods that can be performed in real time. Separating true brain signals from motion artifact could clear the way for EEG brain computer interfaces for assistance during mobile activities, such as walking.

## Introduction

Researchers have made great progress in understanding brain function over the last century, but we still lack information on the complex cortical activity underlying everyday tasks performed by mobile individuals. Studies on immobile subjects have greatly added to our understanding of brain function during cognitive and motor tasks (Näätänen and Picton, 1987; Klimesch, 1999; Pfurtscheller and Lopes da Silva, 1999; Neuper and Klimesch, 2006; Jerbi et al., 2009). Almost all functional brain imaging studies have been limited to lying or seated postures with little body motion. For real world applications like brain machine interfaces and clinical neurorehabilitation, a better understanding of changing brain dynamics during mobile activities like walking would greatly advance current neuroscience knowledge. This rationale has driven researchers in recent years to explore possibilities of electroencephalography (EEG) for mobile brain imaging (Makeig et al., 2009; Gwin et al., 2010, 2011; Gramann et al., 2011, 2014; Presacco et al., 2011; Wagner et al., 2012, 2014; Broccard et al., 2014; Seeber et al., 2014, 2015). However, a current limitation to these efforts is that we do not know how mathematical methods developed for processing EEG data collected on seated or standing subjects will perform on data collected on mobile subjects that will inevitably contain movement artifact.

Due to its portability, low cost, and good time resolution, EEG shows great promise for studying neural activity during mobile tasks. Analysis of brain dynamics during a walking stride requires fine temporal resolution because of its relatively short duration. With millisecond precision, EEG has inherently better temporal resolution than other brain imaging methods such as functional near infrared spectroscopy (Villringer and Chance, 1997; Irani et al., 2007). Blind-source separation methods such as independent component analysis (ICA) combined with inverse modeling of neural sources can provide EEG spatial resolution of approximately 1 cm (Makeig et al., 2004a,b). This combination of good temporal resolution, reasonable spatial resolution, and the low mass of EEG hardware has led to a plethora of new studies on electrocortical activity during human walking (Gramann et al., 2010; Gwin et al., 2010, 2011; Presacco et al., 2011; Debener et al., 2012; Petersen et al., 2012; Severens et al., 2012; Wagner et al., 2012, 2014; Sipp et al., 2013; Kline et al., 2014; Lin et al., 2014; Seeber et al., 2014, 2015; Bulea et al., 2015; Malcolm et al., 2015).

Pervasive, semi-periodic movement artifact is a major drawback of using EEG to examine electrocortical activity during human locomotion (Gwin et al., 2010). Two recent studies have indicated that movement artifact can lead to high levels of spectral power, especially at very low and very high frequencies, during double support (Castermans et al., 2014; Kline et al., 2015). ICA has proven very effective for separating eye and muscle artifacts from EEG electrocortical signals during seated or standing tasks (Jung et al., 2000; Delorme et al., 2007). Algorithms that model independent components as equivalent current dipoles, such as DIPFIT, have also been shown to be able to accurately localize the resultant neural sources (Oostenveld and Oostendorp, 2002). How algorithms like ICA and DIPFIT perform in the presence of the semi-periodic movement artifact inherent to walking is unknown.

Many procedures have been utilized for removing movement artifact during walking. Using a template regression by subtracting a moving average of the 20 surrounding strides and then performing ICA, Gwin et al. (2010) were able to significantly reduce power at lower frequencies (1.5–8.5 Hz) and recover event related potentials for a visual oddball task for walking at 0.8 and 1.2 m/s. More recent research has shown that this method alone or in combination with wavelet filtering does not remove all movement-induced fluctuations from data at speeds from 0.4–1.6 m/s (Kline et al., 2015). More recently, a more sophisticated algorithm, artifact subspace rejection, has been developed (Mullen et al., 2013). This method transforms the data into principal component space and compares the resulting signals to EEG data during quiet standing to identify artifact based on amplitude and variance. However, to function correctly, the thresholds for rejection must be set correctly so that only movement artifact and not neural data are eliminated. Further, Seeber et al. (2015) developed a method to separate overlapping narrow band and broadband frequency activity in EEG. This process, particularly in combination with this group’s method for identifying frequencies that show stride-linked modulation, could be helpful for parsing out neural data during movement.

There are multiple methods available for separating artifacts and determining underlying source locations from EEG data. Many methods stem from blind source separation, which use mathematical algorithms to determine the underlying sources from the EEG data using relatively few assumptions about how the sources were mixed. Of these methods (Bell and Sejnowski, 1995; Belouchrani and Cichocki, 2000; Hyvärinen and Oja, 2000; Lee et al., 2000), adaptive mixture independent component analysis (AMICA) has been shown to be most effective at reducing mutual information between sources (Delorme et al., 2012). Blind source separation methods do not in isolation determine neural location, but can be used in combination with a source localization algorithm, often DIPFIT. Other methods instead focus instead on solving the inverse problem. While there are many methods that can be utilized (Gorodnitsky et al., 1995; Gorodnitsky and Rao, 1997; Grave de Peralta Menendez et al., 1997, 2004; Baillet, 1998; Gençer and Williamson, 1998; Pascual-Marqui, 1999, 2002; Valdes-Sosa et al., 2000; Liu et al., 2005; Schimpf et al., 2005), generally standardized low-resolution brain electromagnetic tomography (sLORETA) has been shown to have the best balance of computational complexity and accuracy (Grech et al., 2008). However, head to head, it remains an open question whether either ICA with DIPFIT or sLORETA performs better. Therefore, due to our group’s previous experience with ICA and DIPFIT, we chose to focus on these algorithms for this current study.

**Figure 1:**
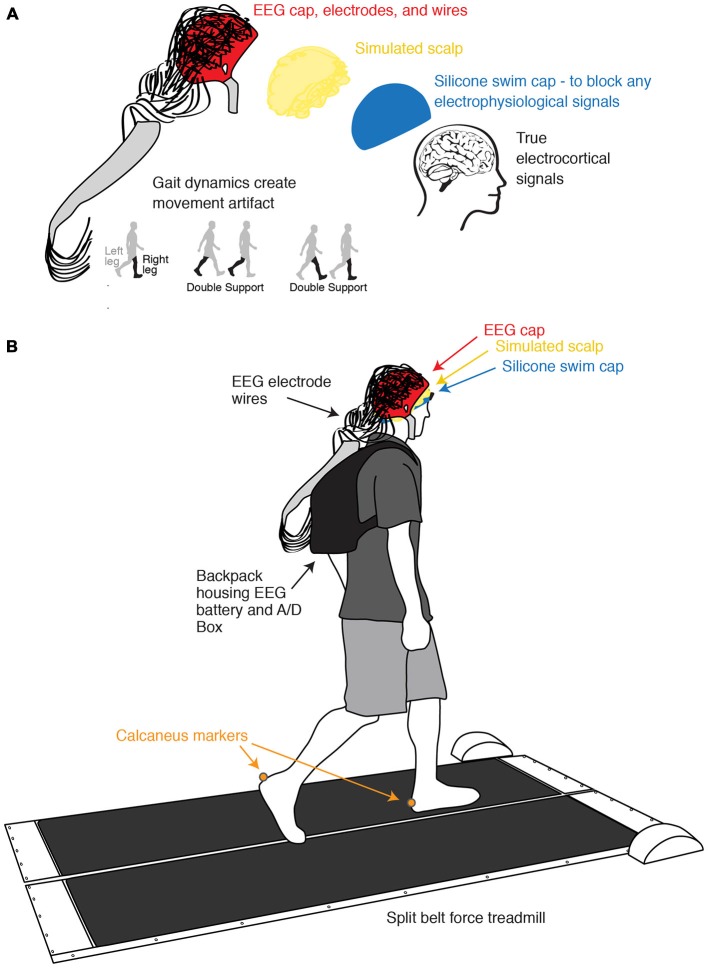
**Experimental setup and channel location illustration. (A)** Illustration of the process of isolating and measuring gait-induced movement artifact in EEG recordings. A simulated conductive scalp permits the electrodes to measure voltage differences resulting from gait dynamics while a silicone swim cap blocks true electrocortical signals. **(B)** Schematic of experimental setup and channel locations. Subjects walked on a custom split-belt force measuring treadmill at four speeds (0.4, 0.8, 1.2, and 1.6 m/s). Calcaneus marker positions were recorded using motion capture.

To test the effect of semi-periodic movement artifact on ICA and dipole fitting, we devised a novel way to measure only gait-related movement artifact with EEG electrodes (Figure 1). We blocked all real electrophysiological signals and collected only movement artifact with an EEG system while ten healthy subjects walked on a treadmill. We applied ICA to this exclusively movement artifact EEG data. If the combination of ICA and DIPFIT was robust to movement artifact, it should find only sources with non-neural locations and characteristics.

## Materials and Methods

### Movement Artifact Data Collection and Initial Processing

Ten subjects participated in the study. All subjects were young and healthy, had no known muscular or neurological deficits, and provided informed consent to the protocol approved by the University of Michigan Internal Review Board before participation. We had the subjects wear a non-conductive silicone swim cap to block all true electrophysiological signals. Over the silicone layer, we placed a simulated scalp with impedance similar to actual human scalp, which consisted of a very short wig soaked in conductive gel. We measured the resistances between the ground and the electrodes on the simulated scalp using a multimeter (0.9 ± 0.4 Mohm). This is within an order of magnitude of the values of real human scalp (Fish and Geddes, 2003). We then set up the EEG system as usual (Figure 1). Subjects walked at four different speeds (0.4, 0.8, 1.2, and 1.6 m/s) for 10 min each while we recorded movement artifact at 512 Hz using a 256-channel active electrode array system (BioSemi Active 2; Amsterdam, Netherlands). We simultaneously recorded kinematics using a 10-camera motion capture system (Vicon Nexus, Oxford, UK) and ground reaction forces using a custom-built, force-instrumented treadmill. Calcaneus marker and ground reaction force data were used to calculate gait events. Specific data collection methods for this portion of the study have been previously reported (Kline et al., 2015).

Our movement artifact processing was similar to previous EEG walking studies (Gwin et al., 2011; Kline et al., 2014). After collection, we first filtered the movement artifact data above a frequency of 1 Hz. We then merged trials for all walking conditions into one data set for each subject.

### Independent Component Analysis

We rejected noisy channels before performing ICA on the merged data sets for each subject. We identified noisy channels using similar methods to previous studies, thresholding channels by standard deviation, correlation with neighboring channels, and kurtosis (Gwin et al., 2011; Sipp et al., 2013; Kline et al., 2014). We modified the standard deviation cutoffs for each subject, rejecting channels with standard deviation values (2.3 ± 0.9) that were clear outliers by visual inspection. We used consistent cutoffs for kurtosis and correlation (Kline et al., 2014). Using these cutoffs only eliminated 10’s of channels. This process left an insufficient sample to channel-squared ratio (18.8 ± 0.1, when 30+ is recommended) to guarantee our ICA algorithm’s convergence. We therefore took a spatially distributed subset of the remaining channels, leaving 125.6 ± 8.2 (range 119–148) channels and a sample to channel-squared ratio of 79.0 ± 8.6. We then performed an ICA on the merged set using the AMICA algorithm. The AMICA algorithm combines infomax and multiple mixture methods to separate EEG signals into maximally independent components fixed in space (Palmer et al., 2006, 2008). AMICA was chosen because it has shown to reduce the shared mutual information more fully than other blind source separation algorithms (Delorme et al., 2012), but the results were virtually identical if the CUDAICA algorithm was substituted for AMICA. We calculated equivalent dipole models for each of the resulting components via the DIPFIT function (Oostenveld and Oostendorp, 2002). For components with residual variance (RV) values less than 15% and dipole locations inside the brain, we calculated topographical maps, power frequency spectra, average time course for a stride, and event-related spectral perturbations (ERSP).

We additionally performed a split-half comparison to examine how reliably AMICA identified movement artifact related independent components with RV’s <15% and consistent, neural locations (Groppe et al., 2009). We split the data into two equal halves. The first set consisted of the first half of the data at each speed, concatenated into a single 20-min data set. The other set consisted of the second half of the data at each speed, concatenated into a single 20-min data set. We performed AMICA on each set of data and compared the locations and RVs of the resulting independent components to those found using the full set of data.

For the spectral analysis, we used EEGLAB’s “spectopo” function, which employs Welch’s power spectral density estimate method. We used a window of length 512 samples (1 s), an fft length of 1028, and no overlap between the windows. For comparison, these same methods were employed in calculating spectra for components in the middle sensorimotor cortex for subjects performing a cognitive task while standing and walking at the same four speeds (Kline et al., 2014).

For the ERSP analysis, we epoched the data from ~0.5 s before to 3 s after right heelstrike. This epoch length was chosen to assure that each epoch captured a full stride plus a sufficient time buffer for spectral calculations even for the slowest speed. We used three cycle Morlet wavelets to compute log spectrograms for each individual stride. We then timewarped all strides so that initial right heel strike, left toe off, left heelstrike, right toe off, and the subsequent right heel strike occurred at the same times. For the ERSP values, we timewarped to the mean of these median values and subtracted the mean spectral power over the stride time at each frequency to calculate only the fluctuations around the mean value (Gwin et al., 2011; Sipp et al., 2013). We again used the same methods to calculate ERSP’s around a cognitive task performed while walking at the same four speeds (Kline et al., 2014).

We additionally analyzed how average correlation and mutual information across channels (or components) changed over the analysis process for both artifact data and for data from subjects performing a cognitive task while standing and walking (Kline et al., 2014). We used methods consistent with Delorme et al. (2012) to find the mean mutual information between different channel pairs by averaging first over channel pairs and then subjects. The mutual information:

$$M_{ij}\text{ = }h\text{(}x_{i}\text{) + }h\text{(}x_{j}\text{) - }h\text{(}x_{i}\text{, }x_{j})$$

where *h*(*x_i_*) represents the entropy of the time series of a random variable *x_i_*. We used the typical binning method with a fixed number of bins to create histograms followed a simple Riemann approximation of the integrals to approximate these entropies. Specific details of these methods can be found in the “Methods” section at the end of Delorme et al. (2012).

We compared the mean mutual information between disparate pairs of channels at four different stages: 256 channels of raw data with a common reference; 256 channels of data re-referenced to the average; just the channels that went into the ICA re-referenced to their average; and the component data. There was some increase with speed in common information shared between non-referenced channels for the artifact data, whereas we found no difference with speed in the EEG data. We consequently show different speeds for the artifact data, but not the EEG data.

## Results

**Figure 2:**
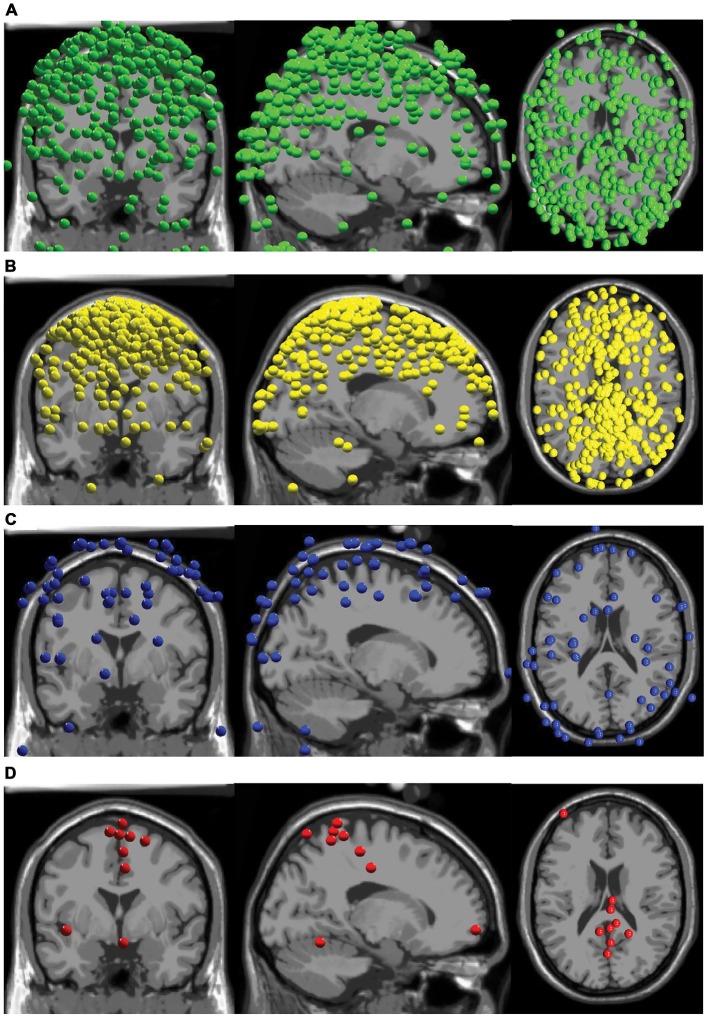
**Component locations. (A)** All components with RVs > 15% with locations outside the brain are shown in green. **(B)** All components with RVs > 15% with locations inside the brain are shown in yellow. **(C)** All components with RVs < 15% with locations outside the brain are shown in blue. **(D)** All components with RVs < 15% with locations inside the brain are shown in red.

Our AMICA results revealed few components that displayed neural characteristics. The components did not have the combination of low RVs, superficial cortical locations, clear dipolar topographical maps, and clean neural spectra commonly displayed by neural sources. Over all subjects, there were a total of 72 components with RVs less than 15%, and 63 of these sources were located outside the brain (Figure 2). There were nine components from as many subjects with RVs lower than 15% in the brain, eight of which were in the cortex (Figure 2).

**Figure 3:**
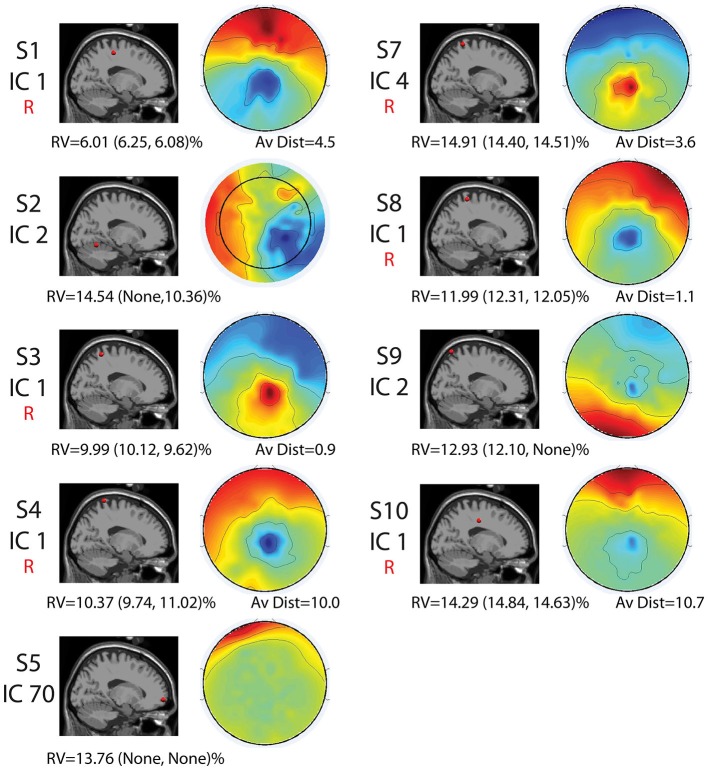
**Cortical component characteristics.** Cortical locations, topographic maps, residual variance (RV) values for the full set and for each half set for the split-half comparisons (in parentheses), and average distance between analogous components for the full-set and each half set in Talairach coordinates are shown for the nine components with RV < 15% and neural locations for the full set. The word “None” appears when there were no components with neural locations and RVs < 15% for a given set. Components that were reliably identified for all three sets are labeled with a red “R.” Alone, these characteristics are not enough to declare all of these components non-neural.

Seven of the nine components with RVs below 15% and locations in the brain shared similar locations and topographical characteristics (Figure 3). However, two revealed differences in location and topographical map characteristics from the other seven. These seven sources were generally located along the midline in the sensorimotor and parietal areas (Figures 2, 2, 3). They displayed topographical maps that appeared somewhat dipolar, but possessed asymmetries and abnormalities that are not typical of true neural sources.

The two remaining sources revealed locations in the cerebellum (Subject 2, Component 2) and the very front of the cortex (Subject 5, Component 70) and maps that did not possess the symmetric, circular pattern typical of dipolar components (Figure 3).

**Figure 4:**
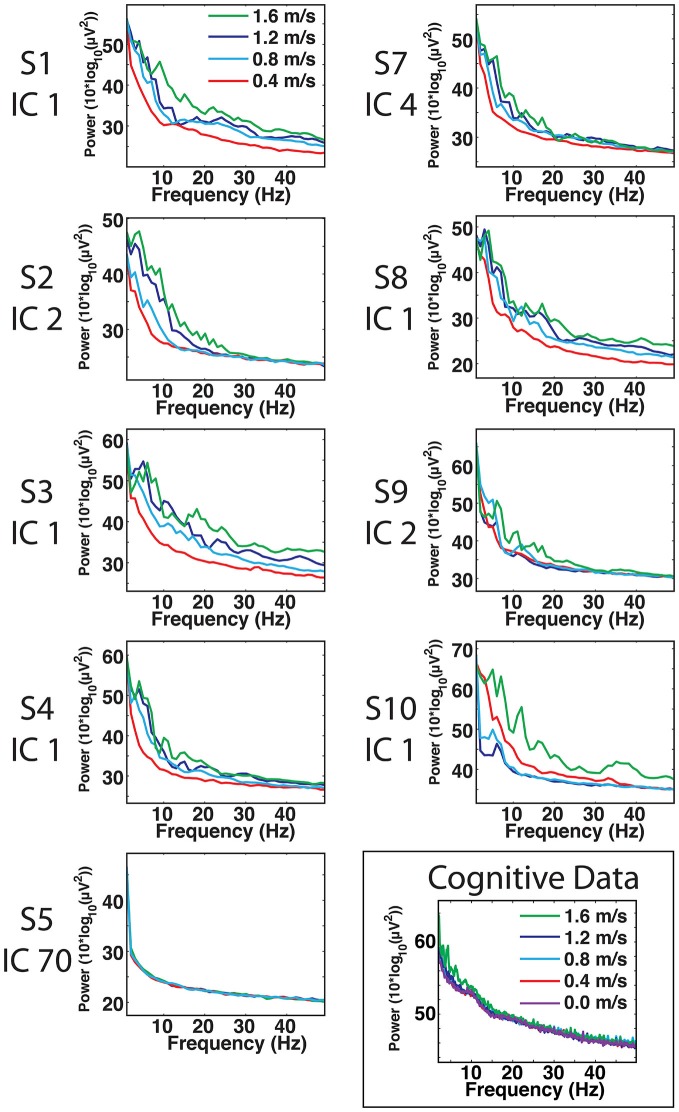
**Cortical component spectra.** Power spectra for the artifact data show large spectral peaks at the stride frequency and resonant frequencies thereof, particularly at speeds of 1.2 and 1.6 m/s. The peaks of the movement artifact data are large compared to those found in neural data, but neural data at 1.6 m/s does show some signs of movement contamination.

**Figure 5:**
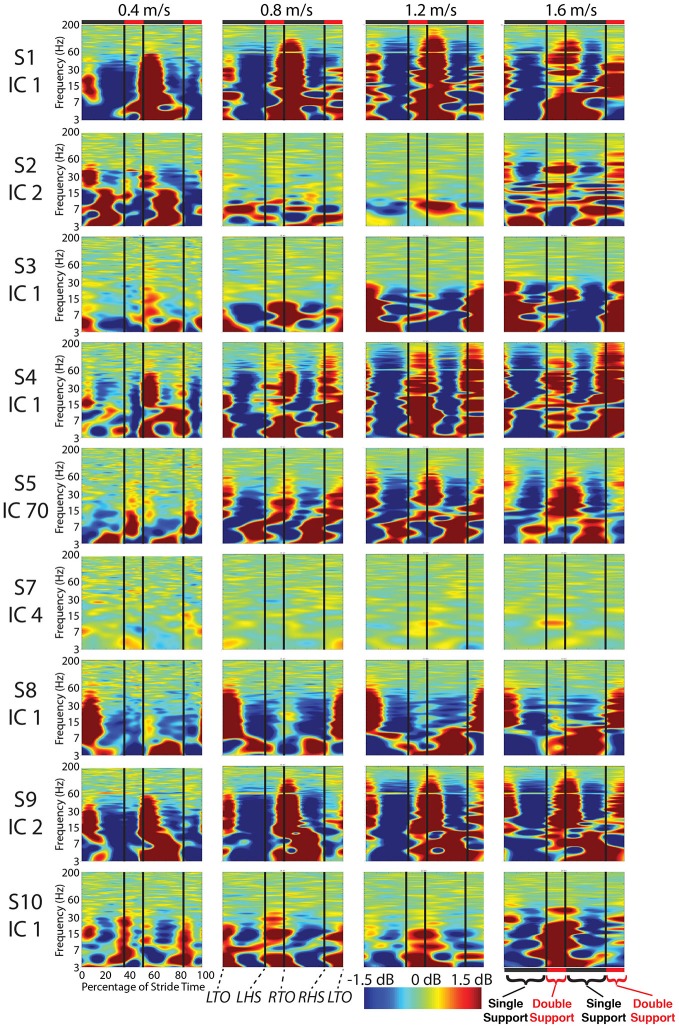
**Cortical component event related spectral perturbations.** Event related spectral perturbations for the nine components with RVs < 15% and locations in the cortex show signs of movement artifact such as large broadband spectral fluctuations. Vertical lines indicate median gait event times for all subjects for all speeds for: left toe-off, left heel strike, right toe-off, and right heel strike.

**Figure 6:**
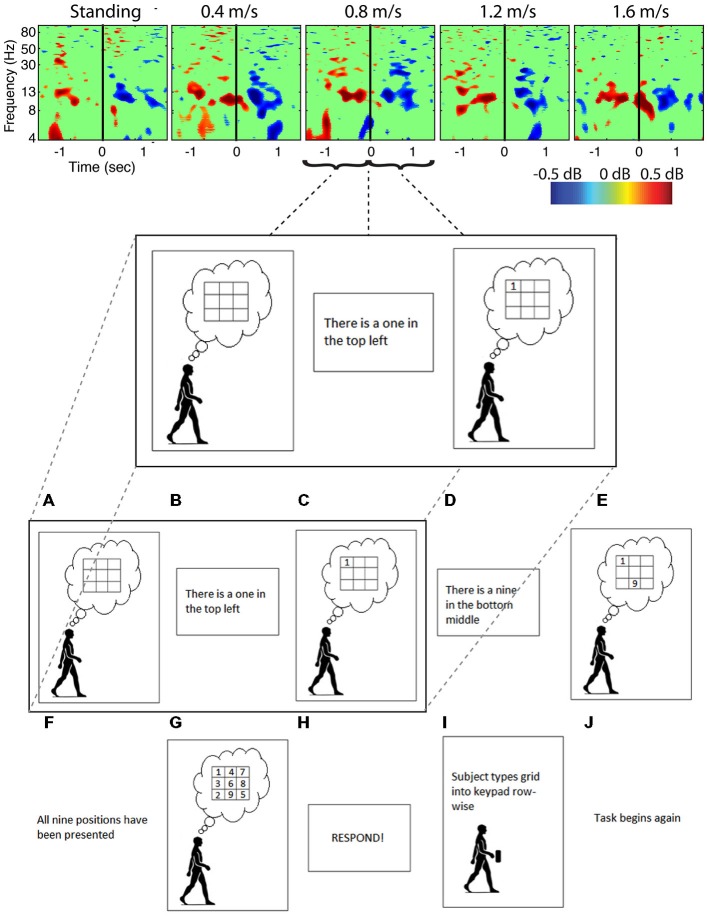
**Cognitive event related spectral perturbations.** Spectral perturbations for subjects performing a Brooks spatial memory task show much smaller fluctuations within a smaller frequency band than the artifact data. Data was epoched around the stimulus (number) presentation, with solid vertical lines indicating the time the stimulus was presented.

For most components, the spectral power and event related spectral perturbations revealed evidence of movement artifact (Figures 4–6). Spectral power showed artifact in the form of peaks at approximately the resonant frequencies of the step frequency (~2 Hz for 1.6 m/s; ~1.8 Hz for 1.2 m/s; 1.5 for 0.8 m/s; 1 Hz for 0.4 m/s). These peaks were more prominent at faster speeds, such as 1.2 and 1.6 m/s, than at slower speeds (Figure 4). In all cases, these spectral peaks were large compared to the changes found in neural components, though the neural data at 1.6 m/s seems to reveal some contamination (Figures 5, 5, 6). The ERSPs generally revealed broadband synchronizations and desynchronizations. The ERSPs also showed generally consistent patterns within a subject across speeds, though there were minor changes as speed increased (Figure 5).

The pattern of shared information reduction over analysis differed for artifact data than for true neural data, and neural data during walking was far more similar to neural data during standing than to artifact data (Tables 1, 2). Much of the common information across channels for the artifact data was eliminated by re-referencing to the average, whereas this re-referencing increased common information for the cognitive data. Despite this reduction in information due to re-referencing, ICA still reduced correlation and mutual information by almost an order of magnitude for the artifact data. For the cognitive data, both eliminating noisy channels and ICA led to a significant reduction in shared information.

**Table 1:** **Mutual information and correlation at various stages of analysis for the artifact data for individual speeds**.

Mutual information (m/s)	Raw data	Average ref	Pre ICA (channels)	Post ICA (components
0.4	0.38	0.046	0.044	0.0105
0.8	0.45	0.064	0.060	0.0113
1.2	0.53	0.074	0.069	0.0121
1.6	0.61	0.090	0.086	0.0123
**Correlation**
0.4	0.64	0.15	0.15	0.017
0.8	0.71	0.21	0.21	0.027
1.2	0.76	0.24	0.23	0.030
1.6	0.78	0.27	0.27	0.032

**Table 2:** **Mutual information and correlation at various stages of analysis for the EEG data for just walking, walking with a cognitive task, and standing with a cognitive task**.

Cognitive data				
Mutual Information	Raw data	Average ref	Pre ICA (channels)	Post ICA (components)
Walking	0.26	0.44	0.23	0.016
Cog walking	0.29	0.36	0.22	0.016
Cog standing	0.27	0.39	0.23	0.016
**Correlation**
Walking	0.42	0.47	0.29	0.019
Cog walking	0.42	0.47	0.30	0.018
Cog standing	0.4	0.47	0.31	0.016

The split-half comparison revealed that cortically located movement artifact components with RV’s < 15% were reliably identified for about half the subjects. AMICA identified reliable components with RV’s of <15% and consistent neural locations for Subjects 1, 3, 4, 7, 8, and 10. For these subjects, components were found for all sets that had an average distance (as measured in Talairach coordinates) between components of 11 Talairach units or less and RV’s of less than 15%. For Subjects 2, 5, 6, and 9, either one or more sets revealed no components with RV’s of <15%.

## Discussion

We performed ICA and dipole fitting on data collected using a novel technique that blocks neural signals and records only pure gait-related artifact. Our ICA and inverse head modeling results identified 99% of components as not being neural based solely on the basis of location and RV above 15%. The remaining 1% of the components had cortical locations and RVs below 15%. These sources were mostly located in cortical areas where one would expect activity during walking. A reliability analysis revealed that these components show consistent locations and low RV’s for about half the subjects. Power spectra and ERSPs of the these components need to be examined to better differentiate some independent components as non-neural sources (Onton et al., 2006). Spectral power peaks at stride frequency and broadband synchronization and desynchronization can help to identify EEG components that are primarily related to movement artifacts rather than electrocortical activity.

The cortically located components were generally found in locations where we would expect neural activity during walking, such as the sensorimotor and posterior parietal cortices. Neural activity has been found previously in the sensorimotor and posterior parietal cortices both in EEG and fNIRS studies during human locomotion (Suzuki et al., 2008; Kurz et al., 2012; Wagner et al., 2012, 2014; Sipp et al., 2013; Koenraadt et al., 2014; Gramann et al., 2014; Seeber et al., 2014, 2015). These other observations suggest that there is likely ongoing real electrocortical activity in these locations during human locomotion.

Analysis of power spectra and ERSPs clearly distinguished the cortically located movement artifact components as non-neural despite their neural locations (Figures 4–6). Their power spectra revealed artifact at low frequencies that was particularly identifiable for a normal walking speed, 1.2 m/s, and a fast walking speed, 1.6 m/s (Figure 4). Further, for all speeds, the ERSPs for these components generally demonstrated broadband synchronization and desynchronization patterns that are consistent with movement artifact (Figure 5) rather than cognitive changes (Figure 6). These broadband changes made gait-related artifact components identifiable for all speeds. Components from walking data that exhibit these spectra and ERSP patterns should be identified and excluded from any neural analysis.

A split-half comparison showed that the cortically located movement artifact components with RV’s < 15% identified by AMICA were unreliable in slightly less than half the subjects. Two of the components identified as unreliable had locations in the cerebellum and the very front of the frontal cortex, so this method may provide a consistent, algorithmic method for eliminating movement related artifacts with unlikely neural locations, such as the cerebellum. However, while a split-half comparison was effective at identifying non-neural components for some subjects, AMICA still found independent components with RVs < 15% and reliable neural locations for over half of the subjects. Further, AMICA occasionally split similar components from one set to another, so other algorithms, such as CUDAICA, may be still less effective at identifying these components using reliability measures. Therefore, though a split-half analysis may be used to filter out these components for some subjects, is not sufficient for the identification of cortically located movement artifact components for all data.

There are a number of possibilities for why we find cortical locations when no cortical dipolar sources were present. Cortical locations of the IC’s from the movement artifact data could result from a violation of one of the assumptions necessary for ICA analysis: independence of the source signals. Because the gait artifact related changes in electrode voltage are stride-linked, they likely occur with similar timing. Recent testing in our lab on a motionless phantom head has revealed that, if two spatially disparate sources have as little as 50% temporal overlap in activity, ICA with DIPFIT will locate a single source with a spatial location in between the two actual sources. Consequently, if we have movement artifact related signals that occur at different locations around the head with similar timing, this could lead to spatial superposition of these locations during the dipole fitting process. This process could make it appear as if there is a dipole within the brain volume, rather than multiple non-dipolar sources outside of it. Other additional sources could be slight cap movements or electrode tensioning. These results were found only for DIPFIT, and results may differ for other source localization models.

The results from ICA and dipole fitting analysis on pure gait-related movement artifact can offer some insights into how to interpret ICAs performed on walking data. Our results indicate that components can be found in cortical areas where activity is expected during walking, even with pure artifact data as an input, and that for about half the subjects, these components can be reliably found across different data sets. However, examination of power spectra and ERSPs can help determine whether sources in these locations are neural or caused by movement artifact.

Previous studies have shown different patterns of spectral perturbations during walking (Gwin et al., 2011; Wagner et al., 2012, 2014; Seeber et al., 2014, 2015). Data from Gwin et al. (2011) show broadband frequency changes over the course of a stride, whereas others have shown more narrow band fluctuations (Wagner et al., 2012, 2014; Seeber et al., 2014, 2015). Additionally, Petersen et al. (2012) showed coherence between the motor cortex and the tibialis anterior at narrow-band frequencies of 8–12 Hz, and 24–40 Hz, approximately 700–200 ms before heelstrike. The 24–40 Hz range matches the results of some studies (Wagner et al., 2012, 2014; Seeber et al., 2014, 2015). The 8–12 Hz range, found predominantly at the more typical rather than the slower walking speeds, overlaps with the results found by Gwin et al. (2011).

Additionally, broadband spectral fluctuations are not specific to movement artifact and can also occur due to neural data (Miller et al., 2014). Broadband fluctuations have been shown at theta, alpha, and beta frequencies in response to postural perturbation (Varghese et al., 2014), and phase-locking of theta and alpha frequencies has been found in memory tasks (Klimesch et al., 2004). This is especially important to consider in light of the differences between the studies in question. There are different speeds, with Gwin et al. combining speeds of 0.8 and 1.25 m/s and Wagner et al. and Seeber et al. having slower speeds ranging from 0.5–0.61 m/s.

There are also different walking conditions. Gwin and colleagues had subjects walking freely on a treadmill, whereas the other studies had subjects walking in a Lokomat. It is quite possible that the different results stem from a source other than movement artifact, such as increased sensory input in subjects walking at more typical walking speeds without a robotic device. Petersen’s results suggest narrow band coherence can be found at speeds of 1–1.1 m/s, as well as slow speeds (0.35 m/s), but did not include the time during heelstrike. The cause of the differences between the study results therefore remains unknown.

Additionally, in recently completed research out of our lab, a phantom head with electrical characteristics similar to a human head with controllable embedded source signals was subjected to walking-like movement with a setup almost identical to that from Gwin et al., and ICA recovered ground truth signals even when the head was moved up to 6 cm fluctuations at frequencies up to 2 Hz (Gwin et al., 2011; Oliveira et al., 2015). These results indicate that ICA is capable of separating walking-like movement artifact from simulated neural signals. It remains to be shown that it performs similarly on actual neural data.

Though our results offer insights into walking data, there are conflicting pieces of evidence as to how compromising gait-related movement artifact is to ICA analysis of neural data. Our ICA results, taken alone, suggest that caution should be exercised with data that contain gait-related movement artifact, but our mutual information offers more insight. Simply re-referencing all channels to the average eliminated most of the shared information in the movement artifact data. Very little additional mutual information reduction occurred due to ICA. This suggests that most movement artifact data is similar across all channels. It also suggests that ICA may not perform as well on this data because, when most of the common information has been removed by re-referencing, the resulting inputs may not appear to be the mixed inputs assumed by the ICA algorithm, leading ICA to perform sub optimally. Further, re-referencing the cognitive data to the average did not reduce shared mutual information for walking or standing data, with or without a cognitive task. If the movement artifact in this data is consistent across channels, as was found for the artifact data, this suggests that little of the common data across channels, even during walking without a cognitive task, seems to be related to gait patterns. However, ICA performed on walking data, especially at fast speeds or over uneven surfaces that induce more head acceleration, produces components with spectral fluctuations consistent with walking-related artifact. It therefore remains an open question as to how neural data containing gait-related movement artifact is separated by ICA.

To determine how ICA parses out neural and movement artifact data, a next step should be combining known neural data with known movement artifact data and analyzing how ICA parses out the two separate contributions. This would consist of collecting clean, seated neural data, collecting pure movement artifact data collected using a setup similar to that used in this study, summing the two resulting signals, and performing ICA on the combined time series. By including both signals, we could determine whether ICA will parse out movement artifact as separate components similar to the ones found in our study or into components containing both neural and movement artifact elements. In practice, this would likely involve varying the relative amplitudes of these two series to determine whether there is a threshold past which gait-related movement artifact becomes problematic.

The best way to fully separate movement artifact without compromising neural data during walking remains an open question in EEG research and may involve a combination of different hardware or software methods. One hardware-based solution consists of interspersing channels collecting pure movement artifact with channels collecting both neural and movement artifact data. Researchers could then interpolate the artifact only channels to calculate artifact alone at the locations that recorded both movement artifact and neural data, and subtract the artifact data out. Additionally, using an inverse-based model, rather than a blind source separation method, may allow for better identification of movement artifact sources that result in fluctuations that overlap in time and/or frequency with those resulting from neural sources. Alternatively, there are ways to combine the software-based noise rejection methods that have been used previously. For instance, Bulea et al. (2015) combined ICA/DIPFIT with artifact subspace rejection to obtain results on how different parts of the brain activate when changing speed. Further, multiple methods developed by Seeber et al. (2014), including gait phase modulation, which determines what particular frequencies are modulated across a stride, and muscle artifact correction, in which PCA methods are used to distinguish muscle from cortical contributions in similar frequency ranges, could be utilized in concert with artifact subspace rejection, particularly its thresholding capabilities, to distinguish between stride linked signals due to cortical activity, artifact, and muscle. Integrating multiple algorithms with ICA could allow for additional artifact identification and rejection. More research needs to be done on these methods in combination to establish the optimal method for distinguishing neural data from artifact in EEG during human walking.

## Author Contributions

All authors collaborated to design the methods of the study. JEK and HJH collected the data. KLS analyzed the data. KLS and DPF interpreted the data. KLS and DPF drafted the manuscript. KLS, JEK, and HJH edited and revised the manuscript.

## Conflict of Interest Statement

The authors declare that the research was conducted in the absence of any commercial or financial relationships that could be construed as a potential conflict of interest.

## References

[B1] Baillet S. (1998). Toward Functional Imaging of Cortical Electrophysiology Markovian Models for the Source Estimation of Magneto/Electroencephalography and Experimental Assessments. Ph.D. thesis. University of Paris-ParisXI, Orsay.

[B2] Bell A. J. Sejnowski T. J. (1995). An information-maximization approach to blind separation and blind deconvolution. Neural Comput. 7, 1129–1159. 10.1162/neco.1995.7.6.1129 7584893

[B3] Belouchrani A. Cichocki A. (2000). Robust whitening procedure in blind source separation context. Electron. Lett. 36, 2050–2051. 10.1049/el:20001436

[B4] Broccard F. D. Mullen T. Chi Y. M. Peterson D. Iversen J. R. Arnold M.. (2014). Closed-loop brain-machine-body interfaces for noninvasive rehabilitation of movement disorders. Ann. Biomed. Eng. 42, 1573–1593. 10.1007/s10439-014-1032-6 24833254 PMC4099421

[B5] Bulea T. C. Kim J. Damiano D. L. Stanley C. J. Park H. S. (2015). Prefrontal, posterior parietal and sensorimotor network activity underlying speed control during walking. Front. Hum. Neurosci. 9:247. 10.3389/fnhum.2015.00247 26029077 PMC4429238

[B59] Castermans T. Duvinage M. Cheron G. Dutoit T. (2014). About the cortical origin of the low-delta and high-gamma rhythms observed in EEG signals during treadmill walking. Neurosci. Lett. 561, 166–170. 10.1016/j.neulet.2013.12.059 24412128

[B7] Debener S. Minow F. Emkes R. Gandras K. de Vos M. (2012). How about taking a low-cost, small and wireless EEG for a walk? Psychophysiology 49, 1617–1621. 10.1111/j.1469-8986.2012.01471.x 23013047

[B8] Delorme A. Palmer J. Onton J. Oostenveld R. Makeig S. (2012). Independent EEG sources are dipolar. PLoS One 7:e30135. 10.1371/journal.pone.0030135 22355308 PMC3280242

[B61] Delorme A. Sejnowski T. Makeig S. (2007). Enhanced detection of artifacts in EEG data using higher-order statistics and independent component analysis. Neuroimage, 34, 1443–1449. 10.1016/j.neuroimage.2006.11.004 17188898 PMC2895624

[B63] Fish R. M. Geddes L. A. (2003). Medical and Bioengineering Aspects of Electrical Injuries, Tucson: Lawyers & Judges Publishing Company, Inc.

[B9] Gençer N. G. Williamson S. J. (1998). Differential characterization of neural sources with the bimodal truncated SVD pseudo-inverse for EEG and MEG measurements. IEEE Trans. Biomed. Eng. 45, 827–838. 10.1109/10.686790 9644891

[B10] Gorodnitsky I. F. George J. S. Rao B. D. (1995). Neuromagnetic source imaging with FOCUSS: a recursive weighted minimum norm algorithm. Electroencephalogr. Clin. Neurophysiol. 95, 231–251. 10.1016/0013-4694(95)00107-a 8529554

[B11] Gorodnitsky I. F. Rao B. D. (1997). Sparse signal reconstruction from limited data using FOCUSS: a re-weighted minimum norm algorithm. IEEE Trans. Signal Process. 45, 600–616. 10.1109/78.558475

[B12] Gramann K. Ferris D. P. Gwin J. Makeig S. (2014). Imaging natural cognition in action. Int. J. Psychophysiol. 91, 22–29. 10.1016/j.ijpsycho.2013.09.003 24076470 PMC3983402

[B13] Gramann K. Gwin J. T. Bigdely-Shamlo N. Ferris D. P. Makeig S. (2010). Visual evoked responses during standing and walking. Front. Hum. Neurosci. 4:202. 10.3389/fnhum.2010.00202 21267424 PMC3024562

[B14] Gramann K. Gwin J. T. Ferris D. P. Oie K. Jung T. P. Lin C. T.. (2011). Cognition in action: imaging brain/body dynamics in mobile humans. Rev. Neurosci. 22, 593–608. 10.1515/RNS.2011.047 22070621

[B6] Grave de Peralta Menendez R. Hauk O. Gonzalez Andino S. Vogt H. Michel C. (1997). Linear inverse solutions with optimal resolution kernels applied to electromagnetic tomography. Hum. Brain Mapp. 5, 454–467. 10.1002/(sici)1097-0193(1997)5:6<454::aid-hbm6>3.3.co;2-1 20408248

[B15] Grave de Peralta Menendez R. Murray M. M. Michel C. M. Martuzzi R. Gonzalez Andino S. L. (2004). Electrical neuroimaging based on biophysical constraints. Neuroimage 21, 527–539. 10.1016/j.neuroimage.2003.09.051 14980555

[B16] Grech R. Cassar T. Muscat J. Camilleri K. P. Fabri S. G. Zervakis M.. (2008). Review on solving the inverse problem in EEG source analysis. J. Neuroeng. Rehabil. 5:25. 10.1186/1743-0003-5-25 18990257 PMC2605581

[B17] Groppe D. M. Makeig S. Kutas M. (2009). Identifying reliable independent components via split-half comparisons. Neuroimage 45, 1199–1211. 10.1016/j.neuroimage.2008.12.038 19162199 PMC3062525

[B18] Gwin J. T. Gramann K. Makeig S. Ferris D. P. (2010). Removal of movement artifact from high-density EEG recorded during walking and running. J. Neurophysiol. 103, 3526–3534. 10.1152/jn.00105.2010 20410364 PMC3774587

[B19] Gwin J. T. Gramann K. Makeig S. Ferris D. P. (2011). Electrocortical activity is coupled to gait cycle phase during treadmill walking. Neuroimage 54, 1289–1296. 10.1016/j.neuroimage.2010.08.066 20832484

[B20] Hyvärinen A. Oja E. (2000). Independent component analysis: algorithms and applications. Neural Netw. 13, 411–430. 10.1016/S0893-6080(00)00026-5 10946390

[B56] Irani F. Platek S. M. Bunce S. Ruocco A. C. Chute D. (2007). Functional near infrared spectroscopy (fNIRS): an emerging neuroimaging technology with important applications for the study of brain disorders. Clin. Neuropsychol. 21, 9–37. 10.1080/13854040600910018 17366276

[B21] Jerbi K. Ossandón T. Hamamé C. M. Senova S. Dalal S. S. Jung J.. (2009). Task-related gamma-band dynamics from an intracerebral perspective: review and implications for surface EEG and MEG. Hum. Brain Mapp. 30, 1758–1771. 10.1002/hbm.20750 19343801 PMC6870589

[B60] Jung T. P. Makeig S. Humphries C. Lee T. W. Mckeown M. J. Iragui V. Sejnowski T. J. (2000). Removing electroencephalographic artifacts by blind source separation. Psychophysiology 37, 163–178. 10.1111/1469-8986.3720163 10731767

[B22] Klimesch W. (1999). EEG alpha and theta oscillations reflect cognitive and memory performance: a review and analysis. Brain Res. Brain Res. Rev. 29, 169–195. 10.1016/s0165-0173(98)00056-3 10209231

[B23] Klimesch W. Schack B. Schabus M. Doppelmayr M. Gruber W. Sauseng P. (2004). Phase-locked alpha and theta oscillations generate the P1–N1 complex and are related to memory performance. Brain Res. Cogn. Brain Res. 19, 302–316. 10.1016/j.cogbrainres.2003.11.016 15062867

[B24] Kline J. E. Huang H. J. Snyder K. L. Ferris D. P. (2015). Isolating gait-related movement artifacts in electroencephalography during human walking. J. Neural Eng. 12:046022. 10.1088/1741-2560/12/4/046022 26083595 PMC4946867

[B25] Kline J. E. Poggensee K. Ferris D. P. (2014). Your brain on speed: cognitive performance of a spatial working memory task is not affected by walking speed. Front. Hum. Neurosci. 8:288. 10.3389/fnhum.2014.00288 24847239 PMC4021146

[B26] Koenraadt K. L. Roelofsen E. G. Duysens J. Keijsers N. L. (2014). Cortical control of normal gait and precision stepping: an fNIRS study. Neuroimage 85, 415–422. 10.1016/j.neuroimage.2013.04.070 23631980

[B27] Kurz M. J. Wilson T. W. Arpin D. J. (2012). Stride-time variability and sensorimotor cortical activation during walking. Neuroimage 59, 1602–1607. 10.1016/j.neuroimage.2011.08.084 21920441

[B28] Lee T. W. Girolami M. Bell A. J. Sejnowski T. J. (2000). Unifying information-theoretic framework for independent component analysis. Comput. Math. Appl. 39, 1–21. 10.1016/s0898-1221(00)00101-2

[B29] Lin Y. P. Wang Y. Jung T. P. (2014). Assessing the feasibility of online SSVEP decoding in human walking using a consumer EEG headset. J. Neuroeng. Rehabil. 11:119. 10.1186/1743-0003-11-119 25108604 PMC4245767

[B30] Liu H. Schimpf P. H. Dong G. Gao X. Yang F. Gao S. (2005). Standardized shrinking LORETA-FOCUSS (SSLOFO): a new algorithm for spatio-temporal EEG source reconstruction. IEEE Trans. Biomed. Eng. 52, 1681–1691. 10.1109/tbme.2005.855720 16235654

[B57] Makeig S. Debener S. Onton J. Delorme A. (2004a). Mining event-related brain dynamics. Trends Cogn. Sci. 8, 204–210. 10.1016/j.tics.2004.03.008 15120678

[B58] Makeig S. Delorme A. Westerfield M. Jung T. P. Townsend J. Courchesne E. Sejnowski T. J. (2004b). Electroencephalographic brain dynamics following manually responded visual targets. PLoS Biol. 2:e176. 10.1371/journal.pbio.0020176 15208723 PMC423146

[B31] Makeig S. Gramann K. Jung T. P. Sejnowski T. J. Poizner H. (2009). Linking brain, mind and behavior. Int. J. Psychophysiol. 73, 95–100. 10.1016/j.ijpsycho.2008.11.008 19414039 PMC2796545

[B32] Malcolm B. R. Foxe J. J. Butler J. S. De Sanctis P. (2015). The aging brain shows less flexible reallocation of cognitive resources during dual-task walking: a mobile brain/body imaging (MoBI) study. Neuroimage 117, 230–242. 10.1016/j.neuroimage.2015.05.028 25988225 PMC5080979

[B33] Miller K. J. Honey C. J. Hermes D. Rao R. P. denNijs M. Ojemann J. G. (2014). Broadband changes in the cortical surface potential track activation of functionally diverse neuronal populations. Neuroimage 85(Pt. 2), 711–720. 10.1016/j.neuroimage.2013.08.070 24018305 PMC4347924

[B34] Mullen T. Kothe C. Chi Y. M. Ojeda A. Kerth T. Makeig S.. (2013). Real-time modeling and 3D visualization of source dynamics and connectivity using wearable EEG. Conf. Proc. IEEE Eng. Med. Biol. Soc. 2013, 2184–2187. 10.1109/EMBC.2013.6609968 24110155 PMC4119601

[B35] Näätänen R. Picton T. (1987). The N1 wave of the human electric and magnetic response to sound: a review and an analysis of the component structure. Psychophysiology 24, 375–425. 10.1111/j.1469-8986.1987.tb00311.x 3615753

[B36] Neuper C. Klimesch W. (2006). Event-Related Dynamics of Brain Oscillations. Amsterdam; Boston: Elsevier.

[B37] Oliveira A. Schlink B. Konig P. Hairston W. D. Ferris D. P. (2015). “Effectiveness of ICA in retrieving EEG target signals during cyclical head movements using a phantom head,” in Annual International Conference of the IEEE Engineering in Medicine and Biology Society IEEE Engineering in Medicine and Biology Society Conference. (Milan, Italy).

[B38] Onton J. Westerfield M. Townsend J. Makeig S. (2006). Imaging human EEG dynamics using independent component analysis. Neurosci. Biobehav. Rev. 30, 808–822. 10.1016/j.neubiorev.2006.06.007 16904745

[B62] Oostenveld R. Oostendorp T. F. (2002). Validating the boundary element method for forward and inverse EEG computations in the presence of a hole in the skull. Hum. Brain Mapp. 17, 179–192. 10.1002/hbm.10061 12391571 PMC6872070

[B64] Palmer J. A. Kreutz-Delgado K. Makeig S. (2006). “Super-Gaussian mixture source model for ICA,” in Lecture Notes in Computer Science, Vol. 3889 eds Rosca J. Erdogmus D. Principe J. C. Haykin S. (Berlin: Springer), 854–861.

[B65] Palmer J. A. Makeig S. Kreutz-Delgado K. Rao B. D. (2008). “Newton method for the ICA mixture model,” in 33rd IEEE International Conference on Acoustics and Signal Processing (Las Vegas, NV: IEEE), 1805–1808.

[B39] Pascual-Marqui R. D. (1999). Review of methods for solving the EEG inverse problem. Int. J. Bioelectromagn. 1, 75–86.

[B40] Pascual-Marqui R. D. (2002). Standardized low-resolution brain electromagnetic tomography (sLORETA): technical details. Methods Find. Exp. Clin. Pharmacol. 24, 5–12. 12575463

[B41] Petersen T. H. Willerslev-Olsen M. Conway B. A. Nielsen J. B. (2012). The motor cortex drives the muscles during walking in human subjects. J. Physiol. 590(Pt. 10), 2443–2452. 10.1113/jphysiol.2012.227397 22393252 PMC3424763

[B42] Pfurtscheller G. Lopes da Silva F. H. (1999). Event-related EEG/MEG synchronization and desynchronization: basic principles. Clin. Neurophysiol. 110, 1842–1857. 10.1016/s1388-2457(99)00141-8 10576479

[B43] Presacco A. Goodman R. Forrester L. Contreras-Vidal J. L. (2011). Neural decoding of treadmill walking from noninvasive electroencephalographic signals. J. Neurophysiol. 106, 1875–1887. 10.1152/jn.00104.2011 21768121 PMC3296428

[B44] Schimpf P. H. Liu H. Ramon C. Haueisen J. (2005). Efficient electromagnetic source imaging with adaptive standardized LORETA/FOCUSS. IEEE Trans. Biomed. Eng. 52, 901–908. 10.1109/tbme.2005.845365 15887539

[B45] Seeber M. Scherer R. Wagner J. Solis-Escalante T. Müller-Putz G. R. (2014). EEG beta suppression and low gamma modulation are different elements of human upright walking. Front. Hum. Neurosci. 8:485. 10.3389/fnhum.2014.00485 25071515 PMC4086296

[B46] Seeber M. Scherer R. Wagner J. Solis-Escalante T. Müller-Putz G. R. (2015). High and low gamma EEG oscillations in central sensorimotor areas are conversely modulated during the human gait cycle. Neuroimage 112, 318–326. 10.1016/j.neuroimage.2015.03.045 25818687

[B47] Severens M. Nienhuis B. Desain P. Duysens J. (2012). Feasibility of measuring event related desynchronization with electroencephalography during walking. Conf. Proc. IEEE Eng. Med. Biol. Soc. 2012, 2764–2767. 10.1109/EMBC.2012.6346537 23366498

[B48] Sipp A. R. Gwin J. T. Makeig S. Ferris D. P. (2013). Loss of balance during balance beam walking elicits a multi-focal theta band electrocortical response. J. Neurophysiol. 110, 2050–2060. 10.1152/jn.00744.2012 23926037 PMC3841925

[B49] Suzuki M. Miyai I. Ono T. Kubota K. (2008). Activities in the frontal cortex and gait performance are modulated by preparation. An fNIRS study. Neuroimage 39, 600–607. 10.1016/j.neuroimage.2007.08.044 17950626

[B50] Valdes-Sosa P. Marti F. Garcia F. Casanova R. (2000). “Variable resolution electric-magnetic tomography,” in Biomag 96, eds Aine C. J. Stroink G. Wood C. C. Okada Y. Swithenby S. J. (New York, NY: Springer), 373–376.

[B51] Varghese J. P. Marlin A. Beyer K. B. Staines W. R. Mochizuki G. Mcilroy W. E. (2014). Frequency characteristics of cortical activity associated with perturbations to upright stability. Neurosci. Lett. 578, 33–38. 10.1016/j.neulet.2014.06.017 24970752

[B55] Villringer A. Chance B. (1997). Non-invasive optical spectroscopy and imaging of human brain function. Trends Neurosci. 20, 435–442. 10.1016/s0166-2236(97)01132-6 9347608

[B52] Wagner J. Solis-Escalante T. Grieshofer P. Neuper C. Müller-Putz G. Scherer R. (2012). Level of participation in robotic-assisted treadmill walking modulates midline sensorimotor EEG rhythms in able-bodied subjects. Neuroimage 63, 1203–1211. 10.1016/j.neuroimage.2012.08.019 22906791

[B53] Wagner J. Solis-Escalante T. Scherer R. Neuper C. Müller-Putz G. (2014). It’s how you get there: walking down a virtual alley activates premotor and parietal areas. Front. Hum. Neurosci. 8:93. 10.3389/fnhum.2014.00093 24611043 PMC3933811

